# Supplementary figures and images for: Is there a bilingual advantage in auditory attention among children? A systematic review and meta-analysis of standardized auditory attention tests
Source: PLoS One. 2024 May 1;19(5):e0299393. doi: 10.1371/journal.pone.0299393 (PMC11062550; doi:10.1371/journal.pone.0299393)

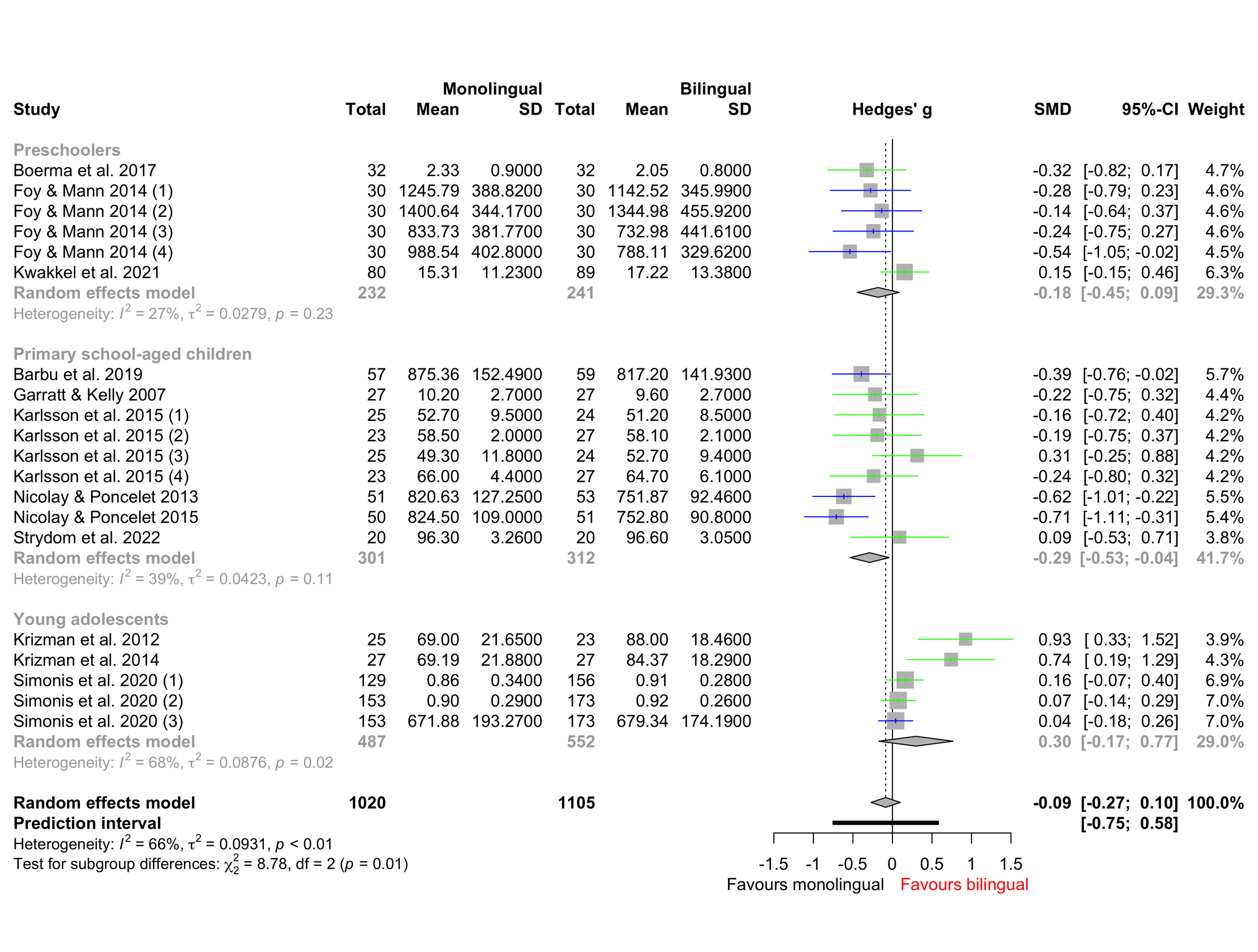

Supplement: S1 Fig — Green lines represent accuracy studies, and blue lines represent RT studies. (TIF) [file pone.0299393.s001.tif]

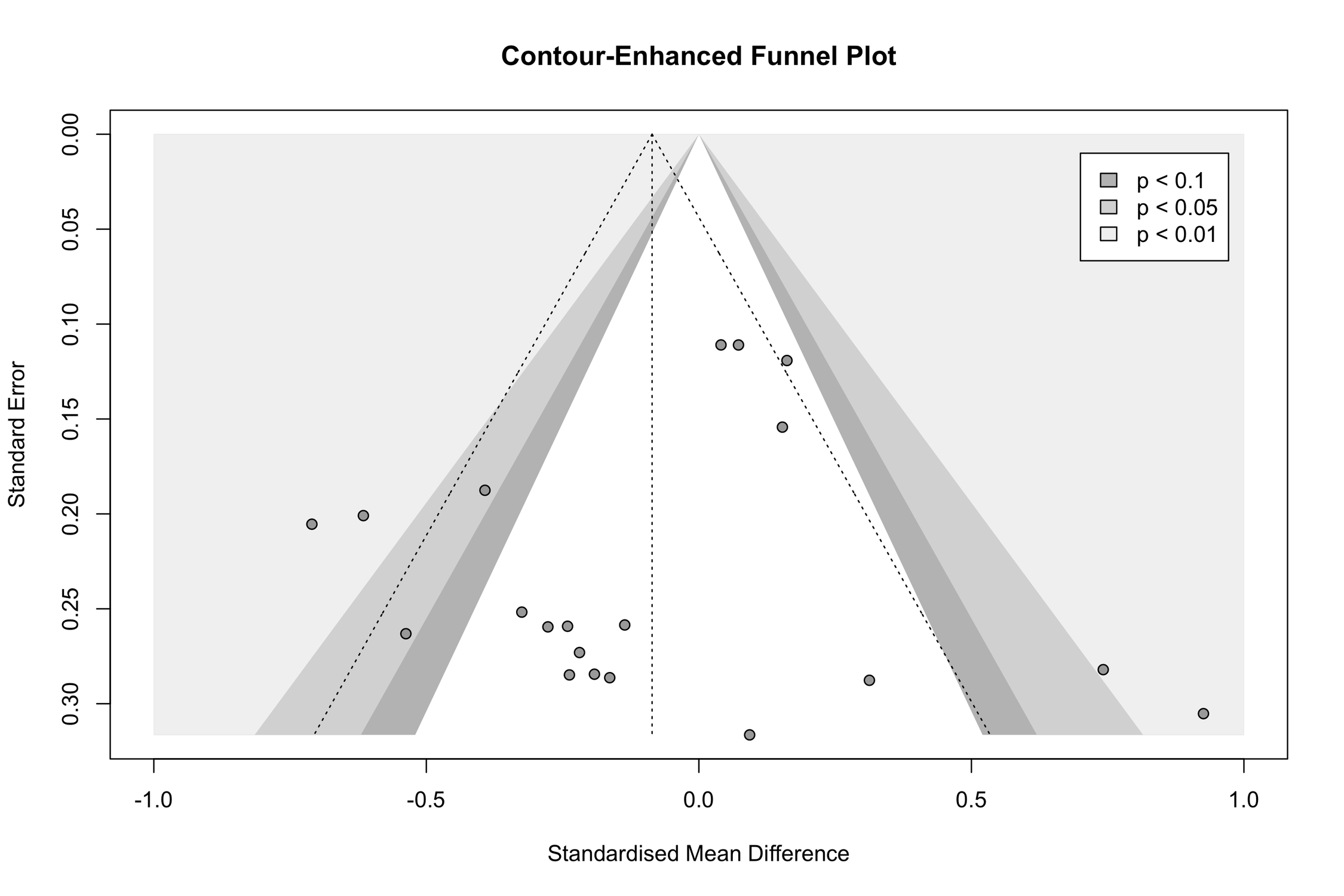

Supplement: S2 Fig — Effect size (i.e., standardized mean difference or SMD) is plotted against its standard error. Each dot represents an individual study. Grey-shaded areas indicate different p-value intervals. (TIF) [file pone.0299393.s002.tif]
