# Supplementary material for: Is there a bilingual advantage in auditory attention among children? A systematic review and meta-analysis of standardized auditory attention tests
Source: PLoS One. 2024 May 1;19(5):e0299393. doi: 10.1371/journal.pone.0299393 (PMC11062550; doi:10.1371/journal.pone.0299393)
Supplement: S1 Table — (DOCX) [file pone.0299393.s003.docx]

**S1** **Table. Search terms used in the electronic databases under the concept of “auditory attention”.**

| OVID Medline | OVID PsycInfo | EBSCO CINAHL |
| --- | --- | --- |
| Auditory Cortex/ | exp Auditory Cortex/ or exp Auditory Evoked Potentials/ or exp Auditory Neurons/ or exp Auditory Masking/ | (MH "Auditory Cortex") OR (MH "Evoked Potentials, Auditory+") |
| Auditory Perception/ | exp Auditory Perception/ or exp Auditory Thresholds/ or exp Auditory Stimulation/ | (MH "Auditory Perception+") |
| Acoustic Stimulation/ | exp Acoustics/ | (MH "Acoustic Stimulation") OR (MH "Speech Acoustics") |
| ((auditor* or hear* or listen*) adj3 attention).tw,kf. | ((auditor* or hear* or listen*) adj3 attention).tw. | TI ( ((auditor* or hear* or listen*) N2 attention) ) OR AB ( ((auditor* or hear* orlisten*) N2 attention) ) |
| ((auditor* or hear* or listen*) adj3 perce*).tw,kf. | ((auditor* or hear* or listen*) adj3 perce*).tw. | TI ( ((auditor* or hear* or listen*) N2 perce*) ) OR AB ( ((auditor* or hear* or listen*) N2 perce*) ) |
| ((acoustic* or auditor* or hear* or listen*) adj3 stimul*).tw,kf. | ((acoustic* or auditor* or hear* or listen*) adj3 stimul*).tw. | TI ( ((acoustic* or auditor* or hear* or listen*) N2 stimul*) ) OR AB ( ((acoustic* or auditor* or hear* or listen*) N2 stimul*) ) |
| (alert* adj3 (auditor* or hear* or listen*)).tw,kf. | (alert* adj3 (auditor* or hear* or listen*)).tw. | TI ( (alert* N2 (auditor* or hear* or listen*)) ) OR AB ( (alert* N2 (auditor* or hear* or listen*)) ) |
| (vigilan* adj3 (auditor* or hear* or listen*)).tw,kf. | (vigilan* adj3 (auditor* or hear* or listen*)).tw. | TI ( (vigilan* N2 (auditor* or hear* or listen*)) ) OR AB ( (vigilan* N2 (auditor* or hear* or listen*)) ) |
| (arous* adj3 (auditor* or hear* or listen*)).tw,kf. | (arous* adj3 (auditor* or hear* or listen*)).tw. | TI ( (arous* N2 (auditor* or hear* or listen*)) ) OR AB ( (arous* N2 (auditor* or hear* or listen*)) ) |
| (orient* adj3 (auditor* or hear* or listen*)).tw,kf. | (orient* adj3 (auditor* or hear* or listen*)).tw. | TI ( (orient* N2 (auditor* or hear* or listen*)) ) OR AB ( (orient* N2 (auditor* or hear* or listen*)) ) |
| (selective attention* adj3 (auditor* or hear* or listen*)).tw,kf. | (selective attention* adj3 (auditor* or hear* or listen*)).tw. | TI ( (selective attention* N2 (auditor* or hear* or listen*)) ) OR AB ( (selective attention* N2 (auditor* or hear* or listen*)) ) |
| (sustained attention* adj3 (auditor* or hear* or listen*)).tw,kf. | (sustained attention* adj3 (auditor* or hear* or listen*)).tw. | TI ( (sustained attention* N2 (auditor* or hear* or listen*)) ) OR AB ( (sustained attention* N2 (auditor* or hear* or listen*)) ) |
| (executive control* adj3 (auditor* or hear* or listen*)).tw,kf. | (executive control* adj3 (auditor* or hear* or listen*)).tw. | TI ( (executive control* N2 (auditor* or hear* or listen*)) ) OR AB ( (executive control* N2 (auditor* or hear* or listen*)) ) |
| (executive attention* adj3 (auditor* or hear* or listen*)).tw,kf. | (executive attention* adj3 (auditor* or hear* or listen*)).tw. | TI ( (executive attention* N2 (auditor* or hear* or listen*)) ) OR AB ((executive attention* N2 (auditor* or hear* or listen*)) ) |
| (target detect* adj3 (auditor* or hear* or listen*)).tw,kf. | (target detect* adj3 (auditor* or hear* or listen*)).tw. | TI ( (target detect* N2 (auditor* or hear* or listen*)) ) OR AB ( (target detect* N2 (auditor* or hear* or listen*)) ) |
