# Supplementary material for: Is there a bilingual advantage in auditory attention among children? A systematic review and meta-analysis of standardized auditory attention tests
Source: PLoS One. 2024 May 1;19(5):e0299393. doi: 10.1371/journal.pone.0299393 (PMC11062550; doi:10.1371/journal.pone.0299393)
Supplement: S2 Table — (DOCX) [file pone.0299393.s004.docx]

**S2** **Table. Search terms used in the electronic databases under the concept of “population”.**

| OVID Medline | OVID PsycInfo | EBSCO CINAHL |
| --- | --- | --- |
| exp Child/ | pediatrics/ | (MH "Child Care") OR (MH "Child+") OR (MH "Child Behavior+") OR (MH "Child Day Care") OR (MH "Child Development") |
| exp Infant/ | childhood development/ or child characteristics/ | (MH "Infant Development") |
| Adolescent/ | adolescent development/ | (MH "Adolescence") OR (MH "Adolescent Behavior") OR (MH "Adolescent Development") |
| (child* or infan* or toddler* or newborn* or neonat* or baby or babies or kid* or p?ediat* or boy* or girl* or pre-pubesc* or prepubesc* or adolesc* or pubescen* or juvenile* or teen* or youth*).tw,kf. | (child* or infan* or toddler* or newborn* or neonat* or baby or babies or kid* or pediat* or paediat* or boy* or girl* or pre pubesc* or prepubesc* or adolesc* or pubescen* or juvenile* or teen* or youth*).tw. | TI ( (child* or infan* or toddler* or newborn* or neonat* or baby or babies or kid* or pediat* or paediat* or boy* or girl* or “pre-pubesc*” or prepubesc* or adolesc* or pubescen* or juvenile* or teen* or youth*) ) OR AB ( (child* or infan* or toddler* or newborn* or neonat* or baby or babies or kid* or pediat* or paediat* or boy* or girl* or “pre-pubesc*” or prepubesc* or adolesc* or pubescen* or juvenile* or teen* or youth*) ) |
| (preschool* or pre-school* or kindergar* or school age or nursery school* or (day care* not adult*) or schoolchild* or elementary school* or secondary school* or middle school* or high school*).tw,kf. | (preschool* or pre school* or kindergar* or school age or nursery school* or schoolchild* or elementary school* or secondary school* or middle school* or high school*).tw. | TI ( (preschool* or “pre- school*” or kindergar* or school age or nursery school* or “day care* or schoolchild* or elementary school* or secondary school* or middle school* or high school*) ) OR AB ( (preschool* or “pre- school*” or kindergar* or school age or nursery school* or “day care* or schoolchild* or elementary school* or secondary school* or middle school* or high school*) ) |
