# Supplementary material for: Is there a bilingual advantage in auditory attention among children? A systematic review and meta-analysis of standardized auditory attention tests
Source: PLoS One. 2024 May 1;19(5):e0299393. doi: 10.1371/journal.pone.0299393 (PMC11062550; doi:10.1371/journal.pone.0299393)
Supplement: S3 Table — (DOCX) [file pone.0299393.s005.docx]

**S3** **Table. Search terms used in the electronic databases under the concept of “study types and methods”.**

| OVID Medline | OVID PsycInfo | EBSCO CINAHL |
| --- | --- | --- |
| Cross Sectional Studies/ |  | (MH "Quantitative Studies") OR (MH "Quasi-Experimental Studies") OR (MH "Experimental Studies") OR (MH "Empirical Research") OR (MH "Repeated Measures") |
| Longitudinal Studies/ |  | (MH "Behavioral Research") OR (MH "Case Studies") OR (MH "Comparative Studies") OR (MH "Descriptive Research") OR (MH "Exploratory Research") OR (MH "Evaluation Research") OR (MH "Methodological Research") OR (MH "Multimethod Studies") OR (MH "Physiological Studies") OR (MH "Pilot Studies") OR (MH "Predictive Research") OR (MH "Replication Studies") OR (MH "Secondary Analysis") OR (MH "Validation Studies") |
| Cohort Studies/ |  | (MH "Prospective Studies") OR (MH "Cross Sectional Studies") OR (MH "Observational Methods") |
| cross sectional.ab,ti. | (cohort or longitudinal).ti,ab,id. or cohort study.md. or longitudinal study.md. | TI ( cross sectional or longitudinal or cohort or observ* ) OR AB ( cross sectional or longitudinal or cohort or observ* ) |
| longitudinal.ab,ti. | (cross section* or observ$).ti,ab. |  |
| (cohort adj (study or studies)).ab,ti. |  |  |
| cohort analy$.ab,ti. |  |  |
| (observ$ adj3 (study or studies)).ab,ti. |  |  |
| ((auditor* or hear* or listen* or shadowing) adj3 (task* or test* or paradigm*)).ab,ti. | ((auditor* or hear* or listen* or shadowing) adj3 (task* or test* or paradigm*)).ab,ti. | TI ( ((auditor* or hear* or listen* or shadowing) N2 (task* or test* or paradigm*)) ) OR AB ( ((auditor* or hear* or listen* or shadowing) N2 (task* or test* or paradigm*)) ) |
| (attention* adj3 (task* or test*)).ab,ti. | (attention* adj3 (task* or test*)).ab,ti. | TI ( (attention* N2 (task* or test*))) OR AB((attention* N2 (task* or test*)) ) |
| ((mapping or cueing or monitoring) adj task*).ab,ti. | ((mapping or cueing or monitoring) adj task*).ab,ti. | TI ( ((mapping or cueing or monitoring) task*) ) OR AB ( ((mapping or cueing or monitoring) task*) ) |
| (task-switching paradigm or dual-task or behavioral or behavioural).ab,ti. | (task-switching paradigm or dual-task or behavioral or behavioural).ab,ti. | TI ( (task-switching paradigm or dual-task or behavioral or behavioural) ) OR AB ( (task-switching paradigm or dual-task or behavioral or behavioural)) |
| (electroencephalography or EEG or event-related potential* or ERP* or evoked potential* or magnetic resonance imaging or MRI or fMRI or near-infrared spectroscopy or NIRS or fNIRS or magnetoencephalography or MEG or positron emission tomography or PET or diffusion tensor imaging or DTI or neuroimaging or brain imaging or brain mapping or electrophysiological or oscillation).ab,ti. | (electroencephalography or EEG or event-related potential* or ERP* or evoked potential* or magnetic resonance imaging or MRI or fMRI or near-infrared spectroscopy or NIRS or fNIRS or magnetoencephalography or MEG or positron emission tomography or PET or diffusion tensor imaging or DTI or neuroimaging or brain imaging or brain mapping or electrophysiological or oscillation).ab,ti. | TI ( (electroencephalography  or EEG or event-related potential* or ERP* or evoked potential* or magnetic resonance imaging or MRI or fMRI or near-infrared spectroscopy or NIRS or fNIRS or magnetoencephalography or MEG or positron emission tomography or PET or diffusion tensor imaging or DTI or neuroimaging or brain imaging or brain mapping or electrophysiological or oscillation) ) OR AB ( (electroencephalography or EEG or event-related potential* or ERP* or evoked potential* or magnetic resonance imaging or MRI or fMRI or near-infrared spectroscopy or NIRS or fNIRS or magnetoencephalography or MEG or positron emission tomography or PET or diffusion tensor imaging or DTI or neuroimaging or brain imaging or brain mapping or electrophysiological or oscillation)) |
| (heart rate or HR or eye-tracking or eye tracking or eye movement* or pupil*).ab,ti. | (heart rate or HR or eye-tracking or eye tracking or eye movement* or pupil*).ab,ti. | TI ( (heart rate or HR or eye-tracking or eye tracking or eye movement* or pupil*) ) OR AB ( (heart rate or HR or eye-tracking or eye tracking or eye movement* or pupil*) ) |
