# Supplementary material for: Is there a bilingual advantage in auditory attention among children? A systematic review and meta-analysis of standardized auditory attention tests
Source: PLoS One. 2024 May 1;19(5):e0299393. doi: 10.1371/journal.pone.0299393 (PMC11062550; doi:10.1371/journal.pone.0299393)
Supplement: S4 Table — (DOCX) [file pone.0299393.s006.docx]

**S4 Table. Random-effects meta-analytic model summary.**

| Number of studies combined: k = 20  Number of observations: o = 2115 | | | | | |
| --- | --- | --- | --- | --- | --- |
|  | SMD | 95%-CI | t | *p*-value |  |
| Random effects model | -0.0858 | -0.2702; 0.0985 | -0.97 | 0.3421 |  |
| Prediction interval |  | -0.7517; 0.5800 |  |  |  |
| Quantifying heterogeneity: | | | | |  |
| tau^2^ = 0.0931 [95%-CI: 0.0338; 0.2908]; tau = 0.3051 [95%-CI: 0.1838; 0.5393] | | | | |  |
| *I*^2^ = 65.8% [95%-CI: 45.1%; 78.6%]; *H* = 1.71 [95%-CI: 1.35; 2.16] | | | | |  |
| Test of heterogeneity: | | | | |  |
| *Q* = 55.51, *df* = 19, *p*-value < 0.0001 | | | | |  |

*k*: number of effect sizes; SMD: standardized mean difference; 95%-CI: 95% confidence interval; tau^2^: estimated amount of residual heterogeneity; tau: square root of estimated tau^2^ value; *I*^2^: residual heterogeneity/unaccounted variability; *H*: the square root of *H*^2^ (i.e., unaccounted variability/sampling variability); *Q*: a weighted sum of squares; *df*: degree of freedom.
