# Supplementary material for: Is there a bilingual advantage in auditory attention among children? A systematic review and meta-analysis of standardized auditory attention tests
Source: PLoS One. 2024 May 1;19(5):e0299393. doi: 10.1371/journal.pone.0299393 (PMC11062550; doi:10.1371/journal.pone.0299393)
Supplement: S5 Table — (DOCX) [file pone.0299393.s007.docx]

**S5 Table. Mixed-effects meta-regression model summary, with test measure as the moderator.**

| Mixed-Effects Model (k = 20; tau^2^ estimator: REML) | | | | | |
| --- | --- | --- | --- | --- | --- |
| tau^2^ = 0.0455 (SE = 0.0311), tau = 0.2133, *I*^2^ = 52.11%, *H*^2^ =2.09, *R*^2^ = 51.13% | | | | | |
| Test of Moderators: *F* (*df*1 = 1, *df*2 = 18) = 9.3759, *p*-value = 0.0067 | | | | | |
| Model Results: | | | | | |
|  | Estimated *g* | Standard Error | *df* | *p*-value | 95%-CI |
| Accuracy | 0.1036 | 0.0943 | 18 | 0.2864 | -0.0945; 0.3017 |
| RTs | -0.4432 | 0.1447 | 18 | 0.0067 ** | -0.7472; -0.1391 |

*R*^2^: amount of heterogeneity accounted for; *g*: effect size.

**p* < 0.05. ***p* < 0.01. ****p* < 0.001.
