# Supplementary material for: Is there a bilingual advantage in auditory attention among children? A systematic review and meta-analysis of standardized auditory attention tests
Source: PLoS One. 2024 May 1;19(5):e0299393. doi: 10.1371/journal.pone.0299393 (PMC11062550; doi:10.1371/journal.pone.0299393)
Supplement: S6 Table — (DOCX) [file pone.0299393.s008.docx]

**S6 Table. Subgroup analysis result, stratified by test measure.**

| Test Measure | *g* | 95%-CI | *I*^2^ | *p* _subgroup_ |
| --- | --- | --- | --- | --- |
| Accuracy | 0.1035 | -0.1158; 0.3229 | 47.6% | 0.0014** |
| RTs | -0.3404 | -0.5696; -0.1112 | 59.1% |  |

**p* < 0.05. ***p* < 0.01. ****p* < 0.001.
