# Supplementary material for: Is there a bilingual advantage in auditory attention among children? A systematic review and meta-analysis of standardized auditory attention tests
Source: PLoS One. 2024 May 1;19(5):e0299393. doi: 10.1371/journal.pone.0299393 (PMC11062550; doi:10.1371/journal.pone.0299393)
Supplement: S7 Table — (DOCX) [file pone.0299393.s009.docx]

**S7 Table. Mixed-effects meta-regression model summary for accuracy studies, with participant age as the moderator.**

| Mixed-Effects Model (k = 12; tau^2^ estimator: ML) | | | | | |
| --- | --- | --- | --- | --- | --- |
| tau^2^ = 0.0128 (SE = 0.0206), tau = 0.1133, *I*^2^ = 21.97%, *H*^2^ =1.28, *R*^2^ = 0.00% | | | | | |
| Test of Moderators: *F* (*df*1 = 1, *df*2 = 10) = 2.7507, *p*-value = 0.1282 | | | | | |
| Model Results: | | | | | |
|  | Estimated *g* | Standard Error | *df* | *p*-value | 95%-CI |
| Intercept | -0.3199 | 0.2691 | 10 | 0.2621 | -0.9195; 0.2798 |
| Participant age | 0.0439 | 0.0265 | 10 | 0.1282 | -0.0151; 0.1030 |
