# Supplementary material for: Is there a bilingual advantage in auditory attention among children? A systematic review and meta-analysis of standardized auditory attention tests
Source: PLoS One. 2024 May 1;19(5):e0299393. doi: 10.1371/journal.pone.0299393 (PMC11062550; doi:10.1371/journal.pone.0299393)
Supplement: S8 Table — (DOCX) [file pone.0299393.s010.docx]

**S8 Table. Mixed-effects meta-regression model summary for accuracy studies, with stimulus type as the moderator.**

| Mixed-Effects Model (k = 12; tau^2^ estimator: ML) | | | | | |
| --- | --- | --- | --- | --- | --- |
| tau^2^ = 0.0000 (SE = 0.0117), tau = 0.0011, *I*^2^ = 0.00%, *H*^2^ =1.00, *R*^2^ = 0.00% | | | | | |
| Test of Moderators: *F* (*df*1 = 1, *df*2 = 10) = 0.0033, *p*-value = 0.9550 | | | | | |
| Model Results: | | | | | |
|  | Estimated *g* | Standard Error | *df* | *p*-value | 95%-CI |
| Linguistic stimuli | 0.1099 | 0.1074 | 10 | 0.3303 | -0.1294; 0.3493 |
| Non-linguistic stimuli | -0.0098 | 0.1691 | 10 | 0.9550 | -0.3865; 0.3670 |
