# Supplementary material for: Is there a bilingual advantage in auditory attention among children? A systematic review and meta-analysis of standardized auditory attention tests
Source: PLoS One. 2024 May 1;19(5):e0299393. doi: 10.1371/journal.pone.0299393 (PMC11062550; doi:10.1371/journal.pone.0299393)
Supplement: S9 Table — (DOCX) [file pone.0299393.s011.docx]

**S9 Table. Mixed-effects meta-regression model summary for accuracy studies, with attention components as the moderator.**

| Mixed-Effects Model (k = 12; tau^2^ estimator: ML) | | | | | |
| --- | --- | --- | --- | --- | --- |
| tau^2^ = 0.0000 (SE = 0.0117), tau = 0.0008, *I*^2^ = 0.00%, *H*^2^ =1.00, *R*^2^ = 0.00% | | | | | |
| Test of Moderators: *F* (*df*1 = 3, *df*2 = 8) = 0.8905, *p*-value = 0.4865 | | | | | |
| Model Results: | | | | | |
|  | Estimated *g* | Standard Error | *df* | *p*-value | 95%-CI |
| Sustained attention | 0.2109 | 0.1140 | 8 | 0.1014 | -0.0519; 0.4737 |
| Executive control | -0.1760 | 0.3059 | 8 | 0.5810 | -0.8814; 0.5295 |
| Selective attention | -0.1360 | 0.1860 | 8 | 0.4855 | -0.5648; 0.2929 |
| Auditory attention overall | -0.4032 | 0.2546 | 8 | 0.1519 | -0.9902; 0.1839 |
