# Supplementary material for: Is there a bilingual advantage in auditory attention among children? A systematic review and meta-analysis of standardized auditory attention tests
Source: PLoS One. 2024 May 1;19(5):e0299393. doi: 10.1371/journal.pone.0299393 (PMC11062550; doi:10.1371/journal.pone.0299393)
Supplement: S10 Table — (DOCX) [file pone.0299393.s012.docx]

**S10 Table. Mixed-effects meta-regression model summary for RT studies, with participant age as the moderator.**

| Mixed-Effects Model (k = 8; tau^2^ estimator: ML) | | | | | |
| --- | --- | --- | --- | --- | --- |
| tau^2^ = 0.0144 (SE = 0.0256), tau = 0.1200, *I*^2^ = 22.57%, *H*^2^ =1.29, *R*^2^ = 63.52% | | | | | |
| Test of Moderators: *F* (*df*1 = 1, *df*2 = 6) = 2.0147, *p*-value = 0.2056 | | | | | |
| Model Results: | | | | | |
|  | Estimated *g* | Standard Error | *df* | *p*-value | 95%-CI |
| Intercept | -0.6882 | 0.2805 | 6 | 0.0496 * | -1.3747; -0.0017 |
| Participant age | 0.0484 | 0.0341 | 6 | 0.2056 | -0.0350; 0.1318 |
