# Supplementary material for: Is there a bilingual advantage in auditory attention among children? A systematic review and meta-analysis of standardized auditory attention tests
Source: PLoS One. 2024 May 1;19(5):e0299393. doi: 10.1371/journal.pone.0299393 (PMC11062550; doi:10.1371/journal.pone.0299393)
Supplement: S11 Table — (DOCX) [file pone.0299393.s013.docx]

**S11 Table. Mixed-effects meta-regression model summary for RT studies, with stimulus type as the moderator.**

| Mixed-Effects Model (k = 8; tau^2^ estimator: ML) | | | | | |
| --- | --- | --- | --- | --- | --- |
| tau^2^ = 0.0392 (SE = 0.0406), tau = 0.1981, *I*^2^ = 50.21%, *H*^2^ =2.01, *R*^2^ = 0.66% | | | | | |
| Test of Moderators: *F* (*df*1 = 1, *df*2 = 6) = 0.4022, *p*-value = 0.5494 | | | | | |
| Model Results: | | | | | |
|  | Estimated *g* | Standard Error | *df* | *p*-value | 95%-CI |
| Linguistic stimuli | -0.2063 | 0.2298 | 6 | 0.4040 | -0.7687; 0.3561 |
| Non-linguistic stimuli | -0.1627 | 0.2566 | 6 | 0.5494 | -0.7907; 0.4652 |
