# Supplementary material for: Is there a bilingual advantage in auditory attention among children? A systematic review and meta-analysis of standardized auditory attention tests
Source: PLoS One. 2024 May 1;19(5):e0299393. doi: 10.1371/journal.pone.0299393 (PMC11062550; doi:10.1371/journal.pone.0299393)
Supplement: S12 Table — (DOCX) [file pone.0299393.s014.docx]

**S12 Table. Mixed-effects meta-regression model summary for RT studies, with attention components as the moderator.**

| Mixed-Effects Model (k = 8; tau^2^ estimator: ML) | | | | | |
| --- | --- | --- | --- | --- | --- |
| tau^2^ = 0.0401 (SE = 0.0411), tau = 0.2003, *I*^2^ = 51.70%, *H*^2^ =2.07, *R*^2^ = 0.00% | | | | | |
| Test of Moderators: *F* (*df*1 = 2, *df*2 = 5) = 0.0631, *p*-value = 0.9396 | | | | | |
| Model Results: | | | | | |
|  | Estimated *g* | Standard Error | *df* | *p*-value | 95%-CI |
| Sustained attention | -0.2589 | 0.2564 | 5 | 0.3590 | -0.9181; 0.4003 |
| Executive control | -0.0756 | 0.3633 | 5 | 0.8433 | -1.0095; 0.8583 |
| Selective attention | -0.1049 | 0.2954 | 5 | 0.7370 | -0.8643; 0.6545 |
