# Supplementary material for: Is there a bilingual advantage in auditory attention among children? A systematic review and meta-analysis of standardized auditory attention tests
Source: PLoS One. 2024 May 1;19(5):e0299393. doi: 10.1371/journal.pone.0299393 (PMC11062550; doi:10.1371/journal.pone.0299393)
Supplement: S13 Table — (DOCX) [file pone.0299393.s015.docx]

**S13 Table. Egger’s regression test result.**

|  | Estimate | Standard Error | *t* value | *p*-value |
| --- | --- | --- | --- | --- |
| Intercept | -0.8770 | 1.0493 | -0.836 | 0.414 |
| X | 0.1116 | 0.2054 | 0.543 | 0.594 |
