# Supplementary material for: Is there a bilingual advantage in auditory attention among children? A systematic review and meta-analysis of standardized auditory attention tests
Source: PLoS One. 2024 May 1;19(5):e0299393. doi: 10.1371/journal.pone.0299393 (PMC11062550; doi:10.1371/journal.pone.0299393)
Supplement: Attachment — Submitted filename: Response to Reviewers.docx [file pone.0299393.s017.docx]

We would like to thank both Reviewers for their constructive comments and insightful feedback. Below is our point-by-point response to each comment.

Reviewer #1

Comment 1.1:

Overall, I think this is an interesting systematic review and meta-analysis of the subject. However, given the small number of papers included in the final analysis, I am not sure all the detailed statistical tests are possible with the sample split in half (only 12 resp. 8 data points analysed with many predictors - why is this division into RT and accuracy studies necessary if you use effect sizes as a dependent variable?). In any case, the non-significance of the results cannot really be interpreted, so the conclusions (that there is no difference) should be tuned down.

Response 1.1:

We really appreciate these important observations. First, regarding the concern about the number of studies included in our meta-analysis, we believe that our sample size (*n* = 20) is sufficient in terms of statistical power. It is not uncommon for published meta-analyses to include a similar number of studies or even less (e.g., Greene, 1997; Huang, 2010; Paulavicius et al., 2020). Also, as a general rule of thumb, subgroup analysis can be conducted when the meta-analysis contains at least 10 studies (Harrer et al., 2021; Schwarzer et al., 2015).

Second, we think that it would be conceptually unwise to include accuracy and RT in the same analysis, even though they are both represented by effect size values. Accuracy and RT correspond to two different aspects of stimuli processing in these auditory attentional tasks: decision signaled by a behavioral response vs. the amount of time it takes to reach the decision. The relationship between the dependent variables associated with these processes are not straightforward; moreover, different patterns of accuracy and RT values have been observed across monolinguals and bilinguals—which is relevant to our study (Morales et al, 2013; Sullivan et al., 2018). Our line of thinking is supported by the statistical analyses reported in the manuscript and highlighted below.

As outlined in the “Results - Meta-analysis” section on page 15, test measure (i.e., accuracy vs. RT) significantly influenced effect size, when all studies (*n* = 20) were considered in constructing mixed-effects meta-regression models. Note that this significance level was much lower than the conventional threshold of 0.05, with a very small *p*-value of 0.0067 (see S4 Table). Furthermore, the fact that test measure was a significant effect moderator was also evidenced by the results of a subgroup analysis: there was a significant difference in effect size between accuracy studies and RT studies (*p* = 0.0014; see S5 Table), with distinct patterns being illustrated in the forest plot in Fig 2.

To summarize, given the between-study heterogeneity significantly affected by test measure, we did not add more variables into the meta-regression model. Otherwise, the pooled effect size won’t be interpreted in a meaningful way. Rather, we split the data into accuracy studies and RT studies and analyzed the effects of other factors in each dataset separately.

Finally, with regards to our conclusion, we had proceeded with caution and wrote that “a bilingual advantage in auditory attention, when measured using standardized tests, likely does not exist or is restricted to certain conditions (e.g., accuracy measure or more proficient bilinguals)” on page 17. To clarify, this conclusion is based on the observed effect size (e.g., *g* = 0.10 among accuracy studies; a positive trend favoring bilingual adolescents), rather than on any *p*-values associated with meta-regression modelling. Further, we also provided two considerations when interpreting our results on the same page: “(1) a very heterogeneous bilingual population was synthesized across studies, wherein bilingualism was poorly assessed; (2) most standardized tests are developed for monolingual English speakers, thus they might not be suitable to measure bilinguals.” Thus, there is probably little room left to further tune down our conclusion.

References:

Greene, J. P. (1997). A meta-analysis of the Rossell and Baker review of bilingual education research. *Bilingual Research Journal, 21*(2-3), 103–122. https://doi.org/10.1080/15235882.1997.10668656

Harrer, M., Cuijpers, P., Furukawa, T. A., & Ebert, D. D. (2021). *Doing Meta-Analysis with R: A Hands-On Guide*. Boca Raton, FL and London: Chapmann & Hall/CRC Press.

Huang, S.-F. (2010). Effects of tasks and glosses on L2 incidental vocabulary learning: Meta-analyses. [Doctoral dissertation, Texas A&M University]. ProQuest Dissertations and Theses Global.

Morales, J., Gómez-Ariza, C. J., & Bajo, M. T. (2013). Dual mechanisms of cognitive control in bilinguals and monolinguals*. Journal of Cognitive Psychology, 25*(5), 531–546. https://doi.org/10.1080/20445911.2013.807812

Paulavicius, A. M., Mizzaci, C. C., Tavares, D. R. B., Rocha, A. P., Civile, V. T., Schultz, R. R., Pinto, A. C. P. N., & Trevisani, V. F. M. (2020). Bilingualism for delaying the onset of Alzheimer’s disease: A systematic review and meta-analysis. *European Geriatric Medicine, 11*, 651–658. https://doi.org/10.1007/s41999-020-00326-x

Schwarzer, G., Carpenter, J. R., & Rücker, G. (2015). *Meta-Analysis with R*. London: Springer.

Sullivan, M. D., Poarch, G. J., & Bialystok, E. (2018). Why is lexical retrieval slower for bilinguals? Evidence from picture naming. *Bilingualism: Language and Cognition, 21*(3), 479–488. https://doi.org/10.1017/S1366728917000694

Minor comments:

Comment 1.2:

The introduction could center a bit more on auditory attention and explain the different paradigms and stimuli used in this domain, so that the partitions used later in the analysis are understandable (p.ex. stimulus type, RT vs accuracy...). By the way, this type of catgorisation of studies should be better described and justified (non-linguistic stimuli for instance seems quite a broad category, why no further breakdown?).

Response 1.2:

Thank you for the feedback. To clarify, instead of addressing auditory attention up front, we approached it step by step in the Introduction. Specifically, we first ushered readers into bilingualism studies on cognition, then narrowed the focus to attention, and finally zeroed in on attention in the auditory domain. Although it may not seem centered on auditory attention at first glance, this “peel-the-onion” way of storytelling serves the purpose of introducing our research better.

Following your suggestion, we have elaborated on the experiment paradigms, outcome measures, and stimuli when mentioning the standardized tests. The revised text reads as follows:

“An initial search in our laboratory indicates that standardized tests are often used to assess children’s auditory attention in research and clinical settings [...]. These tests use different experimental paradigms to target different auditory attention components. For example, the Go/No-Go task is often employed to assess sustained attention, during which participants are asked to respond in some conditions but not to respond in others. Accordingly, depending on the task, different outcome measures are reported, such as response speed and accuracy. In addition, these tests use different types of auditory stimuli, which are either linguistic (e.g., syllables, words) or non-linguistic (e.g., tones, animal sounds; see Results for further information).”

As to the categorization of linguistic vs. non-linguistic stimuli, we did not further break them down. This is because as we explained on page 6, we were interested in whether different stimulus type would affect attentional performance in monolingual and bilingual children. We asked this question given that previous studies have reported mixed results on the bilingualism effects in the non-linguistic domain. Moreover, there is little theoretical motivation that could guide us in terms of how to further break down non-linguistic stimuli. Thus, it is beyond our scope to investigate, for example, whether listening to animal sounds or auditory tones would affect participants’ performance in standardized tests between monolingual and bilingual children.

Comment 1.3:

Furthermore, the discussion of hypotheses why there might be a bilingual advantage (or not) could be developed (p.ex. audio-visual effects in early speech perception....). The dichotomy between simultaneous and sequential bilinguals is not as clear as presented, current amount of exposure/ input seems to play a bigger role and should be at least mentioned (for immersion vs. family contexts, for instance, this would be radically different).

Response 1.3:

Thank you very much for pointing these out. To support our hypothesis about the monolingual vs. bilingual children difference, we have referred to Pons et al. (2015) who demonstrated that bilingual experience modulates early audio-visual speech processing. The revised text reads as follows: “Considering evidence on bilingualism modulating audiovisual speech processing [19], our prediction is that if auditory attention development is shaped by bilingual experience, bilingual children might have more accurate and/or faster responses than their monolingual counterparts in standardized tests.”

To acknowledge the effect of language exposure, we have revised the reasoning of our hypothesis as: “Since the bilingualism effects are more evident among those with higher language proficiency and greater exposure [11, 12], we hypothesize that simultaneous bilinguals (i.e., children who learn both languages before the age of three) would more likely show enhanced auditory attention than sequential bilinguals (i.e., children who learn additional languages after the age of three).”

Comment 1.4:

In the data analysis part, it is not clear how the third reviwer "built consensus", and the model selection by anova vs. AIC/ BIC seems redundant (decide on one criterion, or explain better why they are all needed).

Response 1.4:

Thank you for flagging this to us. To improve clarity, we have revised the sentence as “Afterwards, a third reviewer compared the data extracted by the two reviewers and built consensus: for items where there was a conflict, a final decision was made by selecting or entering the most accurate response. Then the consensus data was exported for analysis.”

To assess model performance, we used the *anova* function of the “metafor” package to determine if one model has a better fit than the other. This function performs a likelihood ratio test; apart from the resulting *p*-value of the test, it provides statistics such as the AIC and BIC values associated with each model, which should also be considered in model comparison (Harrer et al., 2021). In particular, the AIC value, corrected for small samples, penalizes complex models with more predictors to avoid overfitting (Harrer et al., 2021). As we explained in the manuscript, “BIC is preferred over AIC when the heterogeneity is large in the studies [27]”; for both statistics, lower values indicate better model performance. Therefore, it is important to consult multiple statistics rather than solely relying on the *p*-value (see Haysey, 2019).

To make it clearer that we used both criteria in model selection, we have rewritten the relevant text as: “Specifically, we inspected the estimated *p*-value and Akaike’s Information Criterion (AIC) value to assess model performance (Bayesian Information Criterion or BIC is preferred over AIC when the heterogeneity is large in the studies [27]). The full model was favored only when the difference was significant as indicated by the *p*-value (less than the conventional threshold of 0.05) and when it provided a better fit for the data as suggested by lower AIC value.”

References:

Halsey, L. G. (2019). The reign of the *p*-value is over: what alternative analyses could we employ to fill the power vacuum? *Biology Letters, 15*(5). http://doi.org/10.1098/rsbl.2019.0174

Comment 1.5:

Finally, it is not clear whether the bilingual/ monolingual advantages found are corrected for other factors (age/ SES), or whether that was done in the original studies.

Response 1.5:

As we outlined in the eligibility criteria on page 7, “studies that controlled for participants’ age and socio-economic status (SES) were included.” More specifically, age was controlled in all of the original studies, as monolingual and bilingual children were compared within the same age group (see Table 1). In our meta-analysis, to explore the age effects, we included it as a variable of interest in statistical models and found that it did not significantly influence the effect size in accuracy studies and RT studies (see detailed results on page 16).

In addition, we had explicitly stated that monolingual and bilingual children had a comparable SES across all original studies (see Table 1), “except in Simonis et al. [38] where the bilingual group had a higher SES than the monolingual group. Given no significant difference in test performance between the two groups, this article was included in the final analysis” (page 13).

Reviewer #2

General comments: The ms presents the results of a meta-analysis of studies of auditory attention among monolingual and bilingual children. Overall, the ms reads well and presents interesting and informative findings. The following questions and comments should be addressed in a revision.
Response: Thank you very much for the feedback. We have addressed your questions and comments below and revised the manuscript accordingly.

Comment 2.1:
While the authors attempt to distance their study from the larger controversy concerning the bilingual advantage in higher-order cognition (which is probably wise), there are a few places in the ms where they could do better at avoiding unwelcome controversy. First, the authors use evaluative terminology to frame the literature in the Introduction and to describe findings in the Abstract and Discussion. Lines 36-38 in the Abstract, for example, the authors characterize differences between monolingual and bilinguals as relative benefits or advantages. Similarly, evaluative terminology appears in different places in the Introduction (lines 65-75; line 107; etc.), including a prediction on line 107 that bilinguals might perform “better” than monolinguals. What is “better” attention? If the authors do really want to steer clear of the controversy and focus on the science, eliminate evaluative terminology from the discussion of group differences. The authors also (somewhat duplicitously) claim they have no interest in engaging in the debate, but then appeal to Carlson and Meltzoff’s study on line 69 as some sort of incontrovertible evidence of the bilingual advantage. Please look at the C & M findings more closely. They compared 12 (!) bilinguals and 17 (!) monolinguals on 9 measures of EF and found no differences between the groups on any of the 9 measures. Only after correcting for differences in L1 – a highly suspect procedure given known L1 differences between monolingual and bilinguals -- did they find differences on 3 or the 9 EF tasks. As discussed in the literature (see Morton, 2015), this is hardly strong evidence of a group difference, let alone an advantage. Requires some rethinking.

Response 2.1:

We appreciate the points raised by the Reviewer. Indeed, the type of language we use to describe our thoughts is relevant. To minimize evaluative terminology, we have reworded text when addressing group differences, including the Abstract, Introduction, Results, and Discussion sections. Nevertheless, it should be also noted that sometimes it is impossible to avoid evaluative terminology when describing the different outcomes produced by monolinguals and bilinguals. In the tests reviewed here, more accurate and/or faster outcomes are interpreted as “better” performance. For example, in the Abstract, we have revised our findings as: “Specifically, studies reporting accuracy observed marginally greater accuracy in bilinguals (*g* = 0.10), but those reporting response times indicated faster latency in monolinguals (*g* = -0.34).” Similarly, we have changed multiple places in the Introduction. For instance, when presenting the prediction, we have made it clearer by stating that “[...] bilingual children might have more accurate and/or faster responses than their monolingual counterparts in standardized tests”. Also, we have remained more neutral by simply saying that “[...] we acknowledge that bilingualism can exert an influence on cognition at least in some types of bilinguals [...]” (line 69-70), and “[...] the bilingualism effects are more evident among those with higher proficiency and greater exposure [...]” (line 124-125).

In addition, we have deleted the reference of Carlson & Meltzoff (2018) but cited Festman et al. (2022), a fairly recent book chapter which impartially reviewed empirical evidence for confounding and moderating variables (e.g., age of bilingualism, language proficiency, etc.) in bilingual advantage research. We hope this citation represents our stance better.

Comment 2.2:

While the authors should be commended for keeping the Introduction succinct, the motivation for the current analysis and in particular, the predictions, is a bit thin. Line 91, we read that “bilingualism might also affect how auditory attention is allocated in bilingual children.” Beyond the redundant reference to bilingualism in bilingual children, the reader is left wondering why? Is there some theory that would lead to this prediction? How do the predictions and goals described on Page 5 and 6 relate to the theoretical characterization of attention on Page 4 (lines 76-85)? There is no obvious connection.

Response 2.2:

Thank you for the feedback. We acknowledge that our prediction is exploratory in nature, which is based on the evidence from previous bilingualism and attention (mostly visual or audio-visual) research. This actually reflects the status quo of the field: no clear theories exist to explain the monolingual vs. bilingual differences in cognition (Blanco-Elorrieta et al., 2021), and the mechanism is less clear than the evidence for the bilingualism effects (Bialystok, 2017). The assumption given by Bialystok (2017, page 250) is vague too: “Attention is a plausible domain in which to search for a mechanism for the cognitive effects of bilingualism. […] The working hypothesis, therefore, is that lifelong bilingualism impacts a set of processes subsumed under the category of executive attention. Beginning in infancy, the attention system is adapted to the particular demands of a bilingual environment, and these adaptations become apparent in cognitive performance across the left span.”

In addition, to bridge between the theoretical account of attention on page 4 and our goal on page 5, we have added that “We are also interested to explore whether the difference would vary by the attention components assessed.” Thank you very much for pointing this out to us.

Comment 2.3:

From the chart in Figure 2, it appears that there are multiple dependencies between effect sizes included in the analysis including different effects from the same study and different effects from different studies from the same lab. The authors need to provide more information about the mixed-effects model they used in the analysis (see page 15) so the readership can evaluative whether these dependencies were properly modelled.

Response 2.3:

Thank you for the feedback. In Figure 2, each study (as indicated by each row in the first column and each line in the forest plot) does represent an independent effect size, and thus there are no multiple dependencies.

To clarify, for studies taken from the same article, we coded them separately when considering participant age, attention components, stimulus type, and outcome measure (which are exactly the four variables included in our mixed-effects models). For example, Karlsson et al. (2015) tested two age groups (7-year-olds, 11-year-olds) via NEPSY-II that targeted different attention components (“Auditory Attention” subtest for selective and sustained attention, “Response Set” subtest for executive control). Thus, as already detailed in Table 1, Karlsson et al. 2015 (1) represents the effect size of the “Auditory Attention” subtest in the younger group and (2) in the older group; (3) represents the effect size of the “Response Set” subtest in the younger group, and (4) in the older group. Similarly, we considered stimulus type and attention components when coding the four studies from Foy and Mann (2014), and outcome measure and attention components when coding the three studies from Simonis et al. (2020).

To reduce confusion, we have elaborated in the “Results – Synthesis” section on page 10 as well as in the footnote of Table 1 that “Numbers following the names signify individual studies from the same article, which considers participant age, test measure, stimulus type, and attention components.”

Comment 2.4:

The authors need to clarify how measuring bilingualism as a continuum will reveal associations with measures of attention that are obscured when bilingualism is defined dichotomously (see line 364). Bear in mind, many studies comparing monolinguals and bilinguals are comparisons of extreme groups, with monolinguals having very little dual language experience and bilinguals having a whole lot. If there are no differences between extreme groups, what is examining the continuum of bilingualism going to reveal? Is the relation between bilingual language experience and attention non-linear, with the largest effects seen for groups with moderate levels of experience and smaller effects for the extreme groups? Also, what even is a continuum of bilingualism? As the authors so eloquently describe on lines 355-363, an English L2 learner from Spain is very different than a Spanish heritage speaker in the US. Is there really a single continuum that captures this variability? Requires further thought.

Response 2.4:

Thank you for the suggestion, along with a series of thought-provoking questions. By treating bilingualism as a continuum, we meant to (1) consider, measure, and report relevant bilingual variables (language use, proficiency, exposure, etc.) more thoroughly, and (2) define and report the inclusion criteria used to recruit bilinguals (rather than simply saying a certain number of bilingual participants were tested). By doing so, it is then possible to capture the nuances within the bilingual population and make a more meaningful comparison between monolinguals and bilinguals. For example, through asking specific questions about the language environment and use, we could tell how an English L2 learner from Spain is different from a Spanish heritage speaker in the US on this spectrum.

We attempted to explain this in the previous paragraph in the manuscript, “[...] bilingualism was poorly assessed and hardly deemed a continuum. We identified two common themes: (1) most language background questionnaires fail to assess bilingualism comprehensively (i.e., including the use of, exposure to, and proficiency of each language; see [32]); (2) bilingual participants are usually recruited as a comparison group to monolinguals, without explaining inclusion criteria.” Therefore, we did not repeat our definition of bilingualism as a continuum in the current paragraph. However, to improve clarity and remind readers, we have revised the relevant text as: “In future studies, bilingualism should be considered as a continuum, and bilingual variables should be appropriately assessed and reported. This will facilitate our understanding of how various facets of bilingualism (e.g., age of acquisition, language proficiency, language exposure, language use, etc.) interact with auditory attention development.”

We agree that some studies have compared extreme groups of monolinguals and bilinguals. Nonetheless, there is also a large body of research that involves sequential bilinguals (e.g., those who may have limited proficiency and exposure to both languages, relative to simultaneous bilinguals). Though the problem is that, as we pointed out, few studies have vigorously reported in detail what type of bilinguals they tested. As a consequence, the bilingualism effects have been mystified by the mixed results in the field. Yet, the effects appear to be more evident among bilinguals with higher proficiency and more exposure. Thus, it is critical to capture the variability of bilingual experience so as to reveal how it interacts with attention (or other cognitive domains).
