# Supplementary material for: Is there a bilingual advantage in auditory attention among children? A systematic review and meta-analysis of standardized auditory attention tests
Source: PLoS One. 2024 May 1;19(5):e0299393. doi: 10.1371/journal.pone.0299393 (PMC11062550; doi:10.1371/journal.pone.0299393)
Supplement: Attachment — Submitted filename: Response to Reviewers.docx [file pone.0299393.s018.docx]

Since no comments were provided by the original first reviewer, we followed the Editor’s reference to the other two reviewers as Reviewer #1 and Reviewer #2, respectively. We thank both of them for their valuable feedback and thoughtful suggestions. Please see our point-to-point response to each comment below.

Reviewer #1

The original ms is stronger than the revised ms. Things are moving in the wrong direction. The primary weakness remains the motivation for the study. This and other concerns are detailed below.

Comment 1.1:

In their ms, the authors claimed that they want to stay clear of the debate concerning a general bilingual advantage, which is fair. However, with the current revision, the authors place themselves squarely in the middle of the debate and they come across as pretty tone-deaf to what is happening in the field. The last 3 or 4 years have witnessed the publication of several very comprehensive meta-analyses that all conclude there is little evidence that children’s language status is related to executive attention, or executive function more broadly, and that the small amount of evidence that does exist has been helped along by publication bias. Rather than give serious consideration to this evidence, the authors pretend like none of this was ever published. The title of their paper provocatively invokes the notion that bilinguals might be advantaged relative to monolinguals. Then in the first paragraph (lines 46 to 53) of the Intro, they effectively claim that decades of research suggest bilinguals are advantaged in “conflict resolution” and executive control tasks relative to monolinguals. This is simply wrong. The conclusion to be drawn from Gunnerud’s and Lowe’s meta-analyses is that there is NO evidence of any difference between monolingual and bilinguals in attention functions or EF more broadly. NONE. That should be starting point. Claiming that they want to steer clear of the debate but then arguing that bilingualism can influence attention function is disingenuous. This is what the argument has been about – and the supporting evidence is pretty weak. RECOMMENDATION: Look to see whether either Gunnerud or Lowe reviewed studies in which children were administered standardized auditory attention measures, and if not, argue that it might be worth examining. Don't argue you want to stay clear of the debate and then hypothesize that bilinguals will be advantaged in attention. That is not steering clear of the debate.

Response 1.1:

Thank you for the feedback. As opposed to the Reviewer’s observation, we were fully aware of the meta-analyses published in the past few years (e.g., Donnelly et al., 2019; Lehtonen, 2018), but please note that most of these meta-analyses are with adults whereas the focus of our manuscript is the developmental population. In the manuscript, we cited two more recent and comprehensive ones that focused on children—Gunnerud et al. (2020) and Lowe et al. (2021)—as examples. We appreciate the Reviewer’s suggestion and have added that “It is worth noting that when addressing attention, nearly all studies included in these meta-analyses have focused on the (audio-)visual domain, and very few measure auditory attention through tools like behavioral tasks or standardized tests.” Meanwhile, we believe that it is crucial to present a full picture, especially when addressing such a controversial topic, to the wide readership of *PLOS ONE*. Since not every reader is familiar with the bilingual advantage hypothesis, we explained this concept in the first paragraph, and provided two examples about conflict resolution and executive control. Note that we also cited Dunabeitia et al. (2014) to illustrate equal performance (in inhibitory tasks) between monolingual and bilingual children.

Furthermore, we referenced Grundy (2020) who used the Bayesian statistical approach and suggested that “when group differences do appear on EF tasks, bilinguals outperform monolinguals far more likely than chance.” Importantly, Grundy (2020, p. 177) pointed out that these findings “highlight the need to determine when, rather than if, bilinguals outperform monolinguals on EF tasks”, which echoes with our statement that it remains unclear when a bilingual advantage occurs, as delineated at the end of the second paragraph in the manuscript.

In addition, we would like to emphasize that we exercised extreme care to remain impartial in describing hypotheses and making claims throughout the manuscript. For example, it is based on empirical evidence on bilingualism modulating audiovisual speech processing (Pons et al., 2015; not on the bilingual advantage per se) that we predicted “if auditory attention development is shaped by bilingual experience, bilingual children might have more accurate and/or faster responses than their monolingual counterparts in standardized tests.” Critically, keeping clear of a dichotomous debate of the bilingual advantage does not mean that we should and we can ignore the bilingualism effects (put it simply, the bilingual advantage ≠ the bilingualism effects). As we already stated in the third paragraph of the paper, “we acknowledge that bilingualism can exert influence on cognition at least in some types of bilinguals, which has been supported by empirical studies …” It would be appreciated if the Reviewer could be more explicit regarding how we could have formed our hypothesis better.

References:

Donnelly, S., Brooks, P. J., & Homer, B. D. (2019). Is there a bilingual advantage on interference-control tasks? A multiverse meta-analysis of global reaction time and interference cost. *Psychonomic Bulletin and Review,* *26*(4), 1122–1147.

Lehtonen, M., Soveri, A., Laine, A., Järvenpää, J., De Bruin, A., & Antfolk, J. (2018). Is bilingualism associated with enhanced executive functioning in adults? A meta-analytic review. *Psychological Bulletin,* *144*(4), 394–425.

Comment 1.2:

Not sure the argument that attention has not been clearly defined is all that persuasive (lines 75). Bialystok has gone to great lengths distinguishing between selective attention, executive attention, and attentional inertia, and has been very clear that the impact of bilingualism on attention function should be confined to executive but not selective attention. Recent findings from developmental studies and meta-analysis suggest there is no difference between monolinguals and bilinguals within any of these domains of attention function but that is besides the point. Attention as a concept has been quite rigorously defined in the literature on bilingualism. The statement on line 75 is therefore dubious. RECOMMENDATION: Conduct a proper review of the definitions of attention that have been put forward by bilingualism researchers (e.g., Bialystok). They are not that vague.

Response 1.2:

Thank you for the recommendation. We had properly reviewed definitions of attention prior to drafting the manuscript, as it has been an important component of our work. Our statement that attention is vaguely defined in the cognitive literature is supported by our search (see Bialystok & Craik, 2022; Styles, 2006).

We had also conducted a thorough search on how attention has been defined by bilingualism researchers, especially by Bialystok. It is clear that how attention was conceptualized in bilingualism literature has been heavily shaped by attention models in psychology (e.g., Enns, 1990; Posner & Petersen, 1990). It is also important to note the difference between bilingualism researchers using/discussing different attention related concepts (e.g., selective attention, executive attention) and clearly defining them. For example, when discussing “selective attention”, Bialystok (1992) related it to selectivity in Enns’s (1990) visual attention scheme, where it represents the highest form of attention that deals with the limitations of the sensory systems and the finite capacity of brain processes, but she did not provide a clear definition about selective attention. Another essential term is “executive attention”. In Bialystok (2017, p. 250), she mentioned that “the notion of executive attention incorporates elements from executive function models and from attention accounts.” Specifically, she used working memory capacity (i.e., a combination of working memory and attention, proposed by Engle and colleagues; see Shipstead et al., 2015) as an equal construct to executive attention. However, she did not define executive attention directly either.

Rather, attention has not been more clearly conceptualized until in a recent article, Bialystok and Craik (2022), which adopted another concept—“attentional control”—to explain the mechanism behind how bilingualism impacts cognition. Attentional control was broadly described as “a repertoire of processing operations that specific tasks and higher-level cognitive functions can utilize to fulfill their various goals” (Bialystok & Craik, 2022, p. 1253), and composed of procedures that either facilitate mental operations (e.g., selection, goal maintenance, coordination, engagement, disengagement) or inhibit mental operations (e.g., interference suppression, response inhibition). Further, Bialystok and Craik (2022, p. 1260) suggested that “lifelong bilingualism has conferred enhanced levels of attentional control to speakers of two or more languages, providing a robust basis for a range of cognitive tasks, including those dependent on executive functions.”

Therefore, prior to Bialystok and Craik (2022), attention as a concept was not rigorously defined in bilingualism research. To make it clearer, we have added in the manuscript that “In bilingualism research, attention was not clearly conceptualized prior to a recent paper by Bialystok and Craik [16]. According to it, lifelong bilingual experience enhances attentional control, which is defined as a repertoire of processing operations that higher-level cognition utilizes to fulfill various goals [16].” Our observation about the bilingualism literature is that researchers tend to use various executive functioning tasks to examine the bilingualism effects, some of which are related to attention, such as the Dimensional Change Card Sort task (e.g., Bialystok & Martin, 2004) and the flanker task (e.g., Sorge et al., 2017). However, it remains a question even to what extent these tasks measure attention along with its components (rather than other overlapping constructs like working memory, etc.) and what aspects of attention they measure exactly. Yet it has been a common practice that researchers interpret their results around certain attentional concepts (e.g., selective attention, executive attention, attention control, etc.) and integrate with other empirical findings. In part, this could have contributed to the accumulating contradictory findings in the field, which sometimes result from inconsistent definitions of a given construct.

That being said, we fully acknowledge the foundational and inspiring work of Bialystok and colleagues in exploring the association between bilingualism and attention, and it is attentional control that lifelong bilingualism impacts (Bialystok & Craik, 2022). Though in our manuscript, we used the attention accounts developed by Posner and colleagues because they have a relatively clear categorization of attention components that are also found to be represented in different brain regions. Further, this account might be more appropriate when we consider components of auditory attention, which is the focus of our paper.

References:

Bialystok, E. (1992). Selective attention in cognitive processing: The bilingual edge. In R. J. Harris (Ed.), *Cognitive processing in bilinguals* (pp. 501-513). North Holland: Elsevier Science Publishers.

Bialystok, E., & Martin, M. M. (2004). Attention and inhibition in bilingual children: evidence from the dimensional change card sort task. *Developmental Science, 7*(3), 325-339.

Bialystok, E., & Craik, F. I. M. (2022). How does bilingualism modify cognitive function? Attention to the mechanism. *Psychonomic Bulletin & Review, 29*, 1246-1269.

Enns, J. T. (1990). Relations between components of visual attention. In J. T. Enns (Ed.), *The development of attention: Research and theory* (pp. 139-158). North Holland: Elsevier Science Publishers.

Shipstead, Z., Harrison, T. L., & Engle, R. W. (2015). Working memory capacity and the scope and control of attention. *Attention, Perception, & Psychophysics, 77*, 1863-1880.

Sorge, G. B., Toplak, M. E., & Bialystok, E. (2017). Interactions between levels of attention ability and levels of bilingualism in children’s executive functioning. *Developmental Science, 20*, e12408.

Styles, E. A. (2006). *The psychology of attention*. Psychology Press.

Comment 1.3:

It is remarkable that in discussing the fact there were no differences between monolinguals or bilinguals in measures of auditory attention, the authors do not draw any parallels between their findings and those of Gunnerud or Lowe. Not even a reference to these papers. Instead, the first paragraph of the Discussion ends with a vague assertion that their findings may contribute to the development of a theory of the bilingual advantage (line 325 – 328). I’m sorry, but what? How will null findings concerning differences between monolinguals and bilinguals contribute to a theory of the bilingual advantage? Are these findings not more consistent with the conclusions of Gunnerud and Lowe (unreferenced) that there are no differences between monolinguals and bilinguals in measures of attention? RECOMMENDATION: Call a spade a spade. According to the Results, there were no differences. The conclusion should be that there is little evidence for differences.

Response 1.3:

Thank you very much for the helpful recommendation. In order to improve clarity and draw a parallel, we have explicitly stated our conclusion based on the results and connected them to the findings of Gunnerud et al. (2020) and Lowe et al. (2021). Specifically, we added the following text in Discussion: “Overall, there was little difference between monolingual and bilingual children’s performance on standardized auditory attention tests. This finding is consistent with those of Gunnerud et al. [7] and Lowe et al. [8], though both of them analyzed tasks focusing on visual attention.”

In addition, we have revised the previous sentence about theory development as “Our work, however, contributes to the field by uncovering the relation between bilingualism and auditory attention.” We hope this revision reflects the significance of our work more accurately.

Comment 1.4:

Still not clear why measuring bilingualism as a continuum will reveal effects that are not revealed by the study of extreme groups (line 374 to 385). This question came up in the last review and remains unaddressed in the current revision.

Response 1.4:

Thank you for the feedback. In Discussion, we emphasized the importance of assessing bilingualism as a continuum in multiple places, because only by doing so, can we capture the nuance within the bilingual populations and provide a fuller picture of the bilingualism effects. As we already explained in the manuscript, evidence suggests that bilingual variables such as language proficiency, age of acquisition can affect cognitive performance (Gollan et al., 2005; Festman et al., 2022; Tse & Altarriba, 2014). “For example, more proficient bilinguals tend to exhibit a positive influence of bilingualism. Consistent with this, we observed a trend of the bilingual advantage toward adolescents relative to younger children, … ” In other words, a dichotomous view of participants as monolingual vs. bilingual overlooks the individual difference, particularly within bilinguals. The story that bilinguals can tell us is probably way richer than that unfolded by simply comparing them with their monolingual counterparts.

Reviewer #2

As the statistical reviewer I will focus on methods and reporting
Response: We greatly appreciate your feedback on our methods and statistical reporting, which has significantly improved the quality of our manuscript.

Major

Comment 2.1:
1) the quality of the studies has not been assessed using a standard tool for observational studies. this needs to be delivered.

Response 2.1:

Thank you very much for the suggestion. To further improve the rigor of our methodology, we have assessed the quality of the studies via the *Standard Quality Assessment Criteria for Evaluating Primary Research Papers: Quality Scoring for Quantitative Studies* (i.e., the “QualSyst” tool; Kmet et al., 2004), and added a new subsection entitled “Quality assessment” under “Materials and methods”.

This added subsection reads as follows: “The methodological quality of the studies was assessed by two independent reviewers using a modified version of the *Standard Quality Assessment Criteria for Evaluating Primary Research Papers: Quality Scoring for Quantitative Studies* (i.e., the “QualSyst” tool; [26]). Specifically, each study was evaluated according to 11 items based on description or reporting of objective, study design, participant selection, participant characteristics, outcome measure, analytic methods, estimate of variance, results, and conclusions, as well as sample size determination and confounding factors control. Each item was scored depending on the degree to which the specific criteria were met (“yes” = 2, “partial” = 1, “no” = 0), and then a summary score was calculated for each study and averaged between reviewers as the final rating. All studies received a score above 0.8, indicating high quality. Further, we used this tool to identify limitations of the reviewed studies and provide recommendations for future research.”

Comment 2.2:
2) the statistical analysis section needs to be restructured, start with the meta analysis, the heterogeneity etc and finish with the meta-regression (but more on all these, specifically in other points).

Response 2.2:

Thank you for the suggestion. We have restructured the “Data analysis” section, which now starts with the meta-analysis, then between-study heterogeneity, and lastly the meta-regression. In particular, we have provided more details regarding how we did the meta-analysis. The revised text reads as follows:

“Apart from a narrative synthesis of included data, we performed a meta-analysis on the pooled effect size using the “meta” package (version 5.2-0) [27] in *R* [28]. Specifically, we extracted raw effect size data in the form of means and standard deviations of the two groups (monolingual and bilingual) from the included studies. In view of insufficient details being reported, we contacted the original investigators to obtain them or used the WebPlotDigitizer tool [29] to extract data from the graphs (e.g., in Krizman et al. [30). As we anticipated considerable between-study heterogeneity, a random-effects model was built using the “metacont” function to pool effect sizes. Given that our effect size data was continuous, we used the restricted maximum-likelihood estimator [31] to calculate the heterogeneity variance *τ*^2^, and the Hedges method to calculate the standardized mean difference (i.e., Hedges’ *g*). To control for uncertainty in heterogeneity estimates, we used Knapp-Hartung adjustments [32] to calculate the confidence interval (CI) around the pooled effect. In terms of quantifying heterogeneity, we reported the *I*^2^ statistic (i.e., percentage of variability not caused by sampling error) [33] along with its confidence intervals, as suggested in [34].”

Comment 2.3:
3) Meta-regression is a stab in the dark usually and is underpowered to detect anything but massive associations (effectively a regression with X observations, where X is the number of available studies). You should discuss this as a major limitation. Even with 60 or 80 studies, it can provide little insight.

Response 2.3:

Thank you for pointing this out to us. We have added on page 11 that “Of note, a limitation of meta-regression is that it describes an observational association across studies rather than a causal relationship; thus its findings should be interpreted with caution [38, 39].”

Comment 2.4:
4) clearly report how you quantified heterogeneity in the methods section. Also report the confidence intervals for I^2 as argued in <http://www.ncbi.nlm.nih.gov/pubmed/17974687>. A simple formula exists in the seminal 2002 Higgins paper that proposed I^2.

Response 2.4:

Thank you for the suggestion. We have explicitly reported how we quantified statistical heterogeneity in the “Data analysis” section. The revised text reads as “In terms of quantifying heterogeneity, we reported the *I*^2^ statistic (i.e., percentage of variability not caused by sampling error) [33] along with its confidence intervals, as suggested in [34].” Additional details about heterogeneity have also been added when explaining the random-effects meta-analytic model as described above.

Further, in the results section, we provided a heterogeneity summary of the random-effects model on page 17 (see full summary in newly added S4 Table in the Supporting Information) and explicitly reported the *I*^2^ and confidence intervals. The relevant text reads as follows: “According to the random-effects model (see S4 Table), the between-study heterogeneity variance was estimated at *τ*^2^ = 0.09 (95% CI: 0.03-0.29), with an *I*^2^ value of 65.8% (95% CI: 45.1-78.6%) that indicates moderate to substantial heterogeneity. Meta-regression modelling was further implemented to address the heterogeneity effect between studies…”. Though it should be noted that for meta-regression models, we reported *I*^2^ without the confidence intervals, as the latter were not readily available in the model summary output in *R*. However, we also reported other statistics like *τ*^2^, *τ*, *H*^2^, etc.

Minor

Comment 2.5:
1) abstract: add some information on methods, random-effects model? how heterogeneity was quantified? was publication bias assessed and how? how was quality of the studies assessed?

Response 2.5:

Thank you for the suggestion. We have added more information on methods in the Abstract. The relevant text reads as “Twenty studies using standardized tests to assess auditory attention in monolingual and bilingual participants aged less than 18 years were identified. We assessed the quality of these studies using a scoring tool for evaluating primary research. For statistical analysis, we pooled the effect size in a random-effects meta-analytic model, where between-study heterogeneity was quantified using the *I*^2^ statistic. No substantial publication bias was observed based on the funnel plot. Further, meta-regression modelling suggests that … .”

Comment 2.6:
2) how were bilingual families defined? one parent bilingual? Both?

Response 2.6:

Thank you for pointing this out. We have clarified on page 7 that “The bilingual group should be raised in bilingual families (i.e., at least one parent speaks another language than English to the child) … ”.

Comment 2.7:
3) Year may be worth considering in bias assessmen: <http://www.ncbi.nlm.nih.gov/pubmed/25988604>. With newer studies we would be more confident.

Response 2.7:

Thank you for pointing this out. Kicinski et al. (2015) included studies with a wide range of publication years, from 1970s to 2000s. However, since nearly all studies included in our review were published in the recent 10 years (except Garratt & Kelly, 2007) and their methodological quality was evaluated as high based on quality appraisal, we did not further consider publication year in bias assessment. We appreciate that you share this paper with us, and will keep it in mind for future meta-analyses that cover much older studies.

Reference:

Kicinski, M., Springate, D. A., & Kontopantelis, E. (2015). Publication bias in meta-analyses from the Cochrane Database of Systematic Reviews. *Statistics in medicine*, *34*(20), 2781-2793. https://doi.org/10.1002/sim.6525

Comment 2.8:
4) clarify that a random effects model was used in the methods section? was it a DerSimonial-Laird RE model with inverse variance weighting? please clarify.

Response 2.8:

Thank you for the suggestion. We have clarified on page 10 that “As we anticipated considerable between-study heterogeneity, a random-effects model was built using the ‘metacont’ function to pool effect sizes. Given that our effect size data was continuous, we used the restricted maximum-likelihood estimator [31] to calculate the heterogeneity variance *τ*^2^, and the Hedges method to calculate the standardized mean difference (i.e., Hedges’ *g*).”

In addition to considering data type, there is another reason why we chose REML over the DerSimonian-Laird estimator. Specifically, it has been found that the DerSimonian-Laird estimator can be biased when the number of studies is small and heterogeneity is high (Harrer et al., 2021; Makambi, 2004). Yet it is very common to find meta-analyses with relatively few studies and high heterogeneity, as in our case.

References:

Harrer, M., Cuijpers, P., Furukawa, T.A., & Ebert, D.D. (2021). *Doing Meta-Analysis with R: A Hands-On Guide*. Boca Raton, FL and London: Chapman & Hall/CRC Press. ISBN 978-0-367-61007-4.

Makambi, Kepher H. 2004. “The Effect of the Heterogeneity Variance Estimator on Some Tests of Treatment Efficacy.” *Journal of Biopharmaceutical Statistics* 14 (2): 439-49.
