# Supplementary material for: Is there a bilingual advantage in auditory attention among children? A systematic review and meta-analysis of standardized auditory attention tests
Source: PLoS One. 2024 May 1;19(5):e0299393. doi: 10.1371/journal.pone.0299393 (PMC11062550; doi:10.1371/journal.pone.0299393)
Supplement: Attachment — Submitted filename: Response to Reviewers.docx [file pone.0299393.s019.docx]

Once again, we would like to thank both reviewers for their valuable feedback, which has significantly improved our paper. In particular, we have addressed the four comments of Reviewer #2 and revised the manuscript accordingly. Please see our point-to-point response below.

Reviewer #2

The paper is much improved. However, there are a few remaining statements that are either inaccurate, highly biased, or confusing. These should be revised.

1. In the Abstract, the authors refer to the “effects” of bilingualism on cognition (line 25). This is misleading. All research in this field is based on cross-sectional between-subjects comparisons, so at best, current knowledge concerns associations between bilingualism and cognition.

Response: Thank you for the feedback. Following the Reviewer’s suggestion, we have revised it as “A wealth of research has investigated the associations between bilingualism and cognition, especially in regards to executive function.”

Nevertheless, it should be noted that many researchers have referred to the “effects” of bilingualism on cognition, which is widely acceptable in the field. Over decades, for example, Bialystok and colleagues have consistently referred to it in myriads of their publications (e.g., Anderson et al., 2018; Bialystok, 1997, 2007, 2021). Likewise, Paap (2023) has referred to it throughout his book, which was suggested by the Reviewer in another comment.

We would also would like to note that not “all research in this field is based on cross-sectional between-subjects comparisons.” Such a view overlooks the great number (although smaller than cross-sectional designs) and value of longitudinal studies in bilingualism research. For instance, in one of the studies we reviewed in the manuscript, Nicoley and Poncelet (2015) followed monolingual and bilingual kindergarten children for three years and investigated the longitudinal effect of bilingual immersion schooling on cognition (also see Woumans et al., 2016). An example of adult study is Sörman et al. (2017), who tested monolingual and bilingual older adults on dual-tasking over six waves from 1988 to 2014.

References:

Anderson, J. A. E., Grundy, J. G., De Frutos, J., Barker, R. M., Grady, C., & Bialystok, E. (2018). Effects of bilingualism on white matter integrity in older adults. *NeuroImage, 167*, 143–150. <https://doi.org/10.1016/j.neuroimage.2017.11.038>

Bialystok, E. (1997). Effects of bilingualism and biliteracy on children’s emerging concepts of print. *Developmental Psychology, 33*(3), 429–440. <https://doi.org/10.1037/0012-1649.33.3.429>

Bialystok, E. (2007). Cognitive effects of bilingualism: How linguistic experience leads to cognitive change. *International Journal of Bilingual Education and Bilingualism, 10*(3), 210–223. <https://doi.org/10.2167/beb441.0>

Bialystok, E. (2021). Cognitive effects of bilingualism: An evolving perspective. In W. S. Francis (Ed.), *Bilingualism across the lifespan: Opportunities and challenges for cognitive research in a global society* (1st ed., pp. 9–28). New York: Routledge.

Paap, K. (2023). *The bilingual advantage in executive functioning hypothesis: How the debate provides insight into psychology’s replication crisis*. New York: Routledge.

Sörman, D. E., Josefsson, M., Marsh, J. E., Hansson, P., & Ljungberg, J. K. (2017). Longitudinal effects of bilingualism on dual-tasking. *PLOS ONE, 12*(12), e0189299. <https://doi.org/10.1371/journal.pone.0189299>

Woumans, E., Surmont, J., Struys, E., & Duyck, W. (2016). The longitudinal effect of bilingual immersion schooling on cognitive control and intelligence. *Language Learning, 66*(S2), 76–91. <https://doi.org/10.1111/lang.12171>

2. Line 26. “Developmental studies reveal different cognitive profiles between monolinguals and bilinguals in (audio)-visual attention tasks.” This is a biased and misleading characterization of the literature. Current meta-analyses clearly indicate there are no differences between monolingual and bilingual children in attention tasks. Also, what is an “(audio)-visual” attention task? Why not “audio-visual”?

Response: Thank you for the feedback. In the Introduction, we reviewed developmental studies that used methods other than behavioral attention tasks, which are the focus of “current meta-analyses,” and suggested different cognitive outcomes between monolinguals and bilinguals. For example, using event-related potentials, Kuipers and Thierry (2015) found that bilingual toddlers display greater attention to speech than monolingual peers. Similarly, using EEG, Nacar Garcia et al. (2018) found that bilingual infants show increased attention to the speech signal than monolinguals. To reflect this, we have specified in the revised sentence that “some” studies revealed differences between monolinguals and bilinguals.

By “(audio)-visual”, we meant visual or audio-visual. To improve clarity, we have revised the relevant sentence as “Some developmental studies reveal different cognitive profiles between monolinguals and bilinguals in visual or audio-visual attention tasks …”

Reference:

Nacar Garcia, L., Guerrero-Mosquera, C., Colomer, M., & Sebastian-Galles, N. (2018). Evoked and oscillatory EEG activity differentiates language discrimination in young monolingual and bilingual infants. *Scientific Reports, 8*, 2770. https://doi.org/10.1038/s41598-018-20824-0

3. Line 367. “Our work…contributes to the field by uncovering the relation between bilingualism and auditory attention.” How does this statement hang together with the sentence on line 360 stating that the bilingual advantage likely does not exist? Based on the results they are reporting, what the authors have uncovered is that there is no relation. Confusing.

Response: Thank you for the feedback. To improve clarity, we have revised the sentence as “Our work specifically suggests that a bilingual advantage is not observable when measured through standardized auditory attention tests.”

4. Line 366. The authors write that there is no theoretical framework in which they can place their findings. Ken Paap just wrote an entire book on the bilingual advantage hypothesis and the replication crisis in psychology. Null findings like the ones reported in this analysis fit very well within the framework discussed in his book. https://www.routledge.com/The-Bilingual-Advantage-in-Executive-Functioning-Hypothesis-How-the-debate/Paap/p/book/9781032310992

Response: Thank you very much for the suggestion. We have referenced Paap’s book and revised the relevant sentence as “Importantly, our result fits well within the theoretical framework discussed in Paap [59], who highlights that the bilingual advantage either does not exist or is restricted to very specific circumstances.”
